# The Cost of Inpatient Care of Schizophrenia in the Polish and Ukrainian Academic Centers—Poznan and Lviv

**DOI:** 10.1007/s40596-014-0198-4

**Published:** 2014-09-13

**Authors:** Tomasz Zaprutko, Elżbieta Nowakowska, Krzysztof Kus, Rostyslav Bilobryvka, Lyudmyla Rakhman, Andrzej Pogłodziński

**Affiliations:** 1 Poznan University of Medical Sciences, Poznań, Poland; 2 Danylo Halytsky Lviv National Medical University, Lviv, Ukraine

**Keywords:** Schizophrenia, Hospitalization, Costs, Pharmacotherapy

## Abstract

**Objective:**

The authors aimed to analyze and compare treatments of schizophrenia in Poznan and Lviv to present the potential differences between Poland and Ukraine in pharmacotherapy and economic availability of medicines, to emphasize the role of academic centers in the effective treatment of schizophrenia, and to raise the awareness of residents about economics and the cost of inpatient care.

**Methods:**

The analysis was based on 307 hospital records of patients treated in 2010 and 2011 and data from the hospital accounting department. Per the inclusion criteria, 108 adult patients (50 in Poznan and 58 in Lviv) were enrolled in the study. Monetary values were converted into euros (EUR) at the rate published by the National Bank of Poland (NBP) on October 9, 2012.

**Results:**

The total cost of schizophrenia treatment in Poznan was EUR 160,489.26, $\overline{x}$ = EUR 3,209.78 per patient, and in Lviv it was EUR 30,943.38, $\overline{x}$ = EUR 533.5 per patient. Treatment schedules differed between Poznan and Lviv, and pharmacotherapy was limited economically, especially in Lviv.

**Conclusion:**

Although the results differ between Poznan and Lviv, the study shows that schizophrenia treatment is expensive in both centers. Differences in the health care systems make use of innovative neuroleptics unavailable especially in Lviv, which may contribute to non-compliance or higher level of relapses. Distinctive efforts to improve therapies should be made and efforts to equalize access to innovative pharmacotherapy should be supported to improve the therapy’s efficacy and the economic values of schizophrenia treatment.

Mental disorders are increasingly often considered in terms of social problems, and mental health is believed to be one of the most important resources of a modern person [1]. The European Commission, in the *Green Paper* prepared in 2005, indicates the mental health of European Union (EU) citizens as one of the conditions to achieve strategic objectives of the European Union policy, such as a significant improvement of the quality of life of the EU’s population, to ensure social justice and tolerance, and to put Europe back on the path to sustained economic stabilization [2].

Changing style and pace of life, coexisting stress, and the pressure of excess duties or job loss put the mental health of many people to trials with which they are unable to cope [1]. An estimated one in four adult Europeans experiences symptoms of one of the mental disorders within a year [2, 3]. Poor mental health of EU citizens, meanwhile, costs the EU approximately 3–4 % of its gross domestic product (GDP), mainly due to lost productivity [2, 4].

Among the mental disorders, schizophrenia is one of the most serious and disabling, and despite marginalization in many social groups, according to the World Health Organization (WHO), schizophrenia affects approximately 24 million people worldwide [5, 6]—in the USA, 2 million adults suffer from this disorder [1]. Schizophrenia is a chronic disorder requiring long-standing treatment and having a significant impact on patients and their families [7]. Its onset generally occurs in early adulthood [1] and, apart from the health consequences, schizophrenia generates high direct and indirect costs, such as the cost of hospitalization, pharmacotherapy, diagnostic tests, and loss of productivity [8]. WHO assesses that direct costs of schizophrenia account for between 1.6 and 2.6 % of the total national health care expenditures in Western countries [9, 10]. Many analyses, as well as our own research, indicate that the majority of expenses are being allocated to cover direct costs related to the need of hospitalization [1, 7, 11]. Studies by other authors point out that the costs of productivity loss are higher than the direct costs. A study conducted in 2002 in the USA concluded that indirect costs amount to US dollars (USD) 32.4 billion versus 22.7 billion of direct medical costs [12, 13]. Data mentioned above confirm that during a global economic crisis, because of the limited budgets allocated for treatment in individual countries and the simultaneously growing social needs of a modern and effective health care, an analysis of treatment costs and offers of cost-effective solutions optimizing the therapy are necessary and should be the subject of numerous discussions. Studies conducted in academic centers guarantee the reliability of obtained results and the promptness of their publication and provide wide access to such knowledge and allow academic centers to be leaders in the development of effective mental health care. Thus, residents can acquire an exceptional experience concerning economics of the treatment of schizophrenia at the academic level.

Here, we present the economic burden of schizophrenia treatment in Poznan (Poland) and Lviv (Ukraine) and compare the percentage of each component of total cost. We compare treatment schedules and present potential differences in economic availability of innovative medicines. Our analysis will be useful not only for health care decision makers but also for clinicians and residents from academic centers and could contribute to academic leadership in terms of acquiring and spreading knowledge. The examination of the costs of schizophrenia in these countries may lead to the cost-effective treatment of schizophrenia in the long run. In addition, the analysis suggests evidence-based solutions that may contribute to the improvement of treatment efficacy while reducing direct and indirect costs in the long term. Furthermore, it is important to consider that inpatient care is covered by the public insurance in both countries. Despite this, patients from Ukraine are frequently obligated to buy their own innovative medicines during hospitalization because of insufficient hospital budgets. Such limitation in the access to new generations of neuroleptics and the lack of a reimbursement system in outpatient care may contribute to the lack of compliance and generally, to worse results of schizophrenia treatment.

Although Ukrainian psychiatric hospitals provide 47,000 beds, only a few of them offer comprehensive treatment with well-organized, non-pharmacological care [14]. Although in Poland, psychiatric clinics provide 32,000 beds [1], there are far more opportunities for special social training, psychoeducation, and other forms of non-pharmacological treatment. Also important, in Poland, in 2009, there were 133 outpatient psychiatric wards [1]. This way of treatment is still being developed, but the scope of the progress of community psychiatry is still insufficient and requires an urgent and substantial reformulation. In Ukraine, community care is in worse condition [14], but due to some efforts of the Ukrainian Psychiatric Associations and international cooperation between Ukrainian psychiatrists and experts from western countries, community psychiatry is being considered as the important point of comprehensive treatment of schizophrenia. Moreover, initiatives like Psychiatric Summer School Ukraine–Poland–Germany can lead to the improvement of psychiatric care, especially in middle- or low-income countries. Such events, supported by governments, are aimed to provide a forum for sharing practice in the development of mental health service in these countries, and academic centers are included in the study and are intended to contribute to increasing the cognizance about state-of-the-art mental health care services. It confirms the need of progress of psychiatric care both in Poland and Ukraine, and therefore, academic hospitals could be the driving forces behind beneficial solutions, which are innovative and applied in numerous countries. Although the development of psychiatric care requires financial support, mental health should be considered as an investment, not only a cost. However, in both countries analyzed in this study, psychiatric care is underfunded. Thus, knowledge acquired by residents concerning economics and the cost of inpatient care of mental disorders may lead to the awareness of the need to optimize the funding of treatment of schizophrenia among future mental health decision makers.

In Brazil, as in Poland, expenditure on mental health in relation to the total health budget has been reduced [12, 15]. In South America, there was a decrease in funding of patients with mental illness from 5.8 to 2.3 % [12, 15], whereas in Poland, a decrease was observed from 3.7 % in 2000 to 3.29 % in 2010 [1]. Such a trend makes inpatient care in Poland under-financed from the state budget, which leads to the debt of health care facilities. Calculations indicate that the National Health Care Fund in Poland covers an average of only 80 % of costs incurred by the mental health care institutions [1]. Similar results were obtained in our own study. The hospital from Poznan received financial support for only up to 75 % of the total costs generated by the analyzed group of patients. The value of a budgetary support was zloty (PLN) 165 per person per day, and it was not sufficient to cover needs (PLN 212 for women and PLN 225 for men without the costs of medicines and diagnostic tests) of the hospital in Poznan. This amount of money was decreasing to PLN 80.85 per person per day for each day of a hospitalization exceeding 70 days. It confirms the need for an urgent modification of the mental health care founding system in Poland and for an increase of the allocated expenditure. In the UK, spending on mental health treatment in the years 2002–2006 increased from 3.4 billion to 4 billion pounds [12, 16]. The UK’s policy conforms to the objectives of the European Union, where it is emphasized that impaired mental health is a source of additional costs and burdens for the state [2]. People responsible for the funding and organization of the health care in many countries should seek to replicate the UK model, where the total costs of schizophrenia treatment were estimated at 6.7 billion pounds annually and direct costs amounted to 2 billion pounds [17]. A similar discrepancy between direct costs and indirect costs was observed in the population of South Korea, where direct costs amounted to USD 540 million compared to USD 3.2 billion of total costs [11]. In Smark’s study [18] conducted in Australia, indirect costs were two times higher than direct costs, but in Germany, indirect costs of schizophrenia treatment constituted to 87 % of total costs [7, 19]. The high costs of schizophrenia treatment are confirmed in the analysis carried out in India by Grover et al. [20, 21]. The scope of economic burden of schizophrenia confirms that analyses concerning the costs of that disorder are useful and valid. Therefore, every way of treatment, which in a long run will contribute to the improvement of effectiveness of treatment of schizophrenia and will lead to the expected reduction of costs associated with schizophrenia, should be treated as a priority by people responsible for psychiatric care and for a proper education of residents aware of the importance of economics related to mental health care.

## Material and Methods

The study concerns the results of pharmacoeconomic analysis of the costs of inpatient treatment in adult patients hospitalized in Karol Jonscher Hospital of Poznan University of Medical Sciences (Poland) and in the Psychiatric Hospital of Lviv National Medical University (Ukraine). The inclusion criteria were as follows: adulthood of patients of both sexes; schizophrenia diagnosed on the basis of the International Classification of Diseases, Tenth Revision (ICD-10); and hospitalization started and completed between January 2010 and January 2011. Patients were excluded from the study if the hospitalization ended with the patient signing out himself/herself or having a different time period for the hospitalization. In the study, 307 hospital records were analyzed: 127 in Poznan and 180 in Lviv. According to the criteria of the study, 108 hospital records were included: 50 in Poland (25 women and 25 men) and 58 in the Ukraine (33 women and 25 men). Permission to analyze patient records was obtained from hospital decision makers in Poznan and in Lviv and from the previous approval of the proper Bioethics Committee. The study conforms with the Act on Protection of Personal Data. Patients were treated anonymously, and each hospital record was analyzed in collaboration with the psychiatrist involved in the study. Furthermore, each sample can be considered as representative for both countries, because analyzed hospital records concerned a wide range of adult patients. Besides, both cities are known to be quite huge medical and social centers, which gather patients from the whole region.

Necessary sensitive information concerning patient metrics, hospitalization length, medicines used, conducted diagnostic tests, and relapses was gathered and stored in a way to ensure its safety and confidentiality. Data concerning hospital procedures used, data from the hospital formulary, and the price list of diagnostic tests were obtained from the hospital accounting departments. The cost was counted for hospital stay, pharmacotherapy, and diagnostic tests separately, and subsequently obtained amounts were added, obtaining the total cost of inpatient care. The cost per day is used in calculating costs of the inpatient care of schizophrenia. In Poznan a cost per day (without medicines and diagnostic tests) is PLN 225 (euros (EUR) 55.28) for women and PLN 212 (EUR 52.09) for men. In Lviv, a cost per day is hryvnia (UAH) 135.30 (EUR 12.82) regardless of the sex of the patient. Cost of stay was obtained by multiplying the length of stay of patient by the cost per day. Medicine prices from 2011 come from the wholesale price list, which, because of the frequent need for individual medicines purchased by patients in the Ukraine, is to ensure comparability of the cost of drug therapy among analyzed countries. Moreover, costs covered individually by Ukrainian patients were included in the pharmacotherapy cost calculation, because these costs are also an integral part of inpatient care. In order to calculate the cost of pharmacotherapy, the exact amount of used medicines was calculated, and then it was converted to the monetary values resulting from the full price of medicines packages, whereas the cost of diagnostic tests was calculated by adding values of performed tests.

Moreover, money values were converted from the PLN and from the UAH to the European currency on the basis of the average euro exchange rate published by the National Bank of Poland (NBP) on October 9, 2012 (EUR 1 = PLN 4.07; EUR 1 = UAH 10.55). Monetary values presented in the study are a rounding of calculated amounts, which results from the conversion of monetary units into the common European currency.

### Statistics

The data are shown as mean values ± SEM. The data distribution pattern was not normal (unlike Gaussian function). Statistical analyses for age in years and hospitalization were carried out using the nonparametric Kruskal-Wallis test for unpaired data.

## Results

In the analyzed group of patients from Poznan, the average age was 33.62 years, whereas patients from Lviv were almost 6 years older (39.26 years) (see Table 1). Statistical difference between Poznan and Lviv was observed (*p* = 0.0242). The average length of hospitalization of the patients from Poznan was $\overline{x}$ = 54.64 days, and in Lviv, $\overline{x}$ = 38.43 days, and statistical difference between Poznan and Lviv was observed (*p* = 0.0006). Women from the Polish center were hospitalized 22 days less than men, using 1,091 and 1,641 hospital days, respectively. In Lviv, however, men were hospitalized 6.5 days fewer than women. In Poland, in relation to women, the shortest and the longest hospitalization lasted 20 and 89 days and, among men, 25 and 116 days, respectively. Meanwhile, in Lviv, these indicators are 10 and 90 days in the female group, and 13 and 64 days among men.

**Table 1:** Structure of the study group

	Total		Women		Men	
	$\overline{\boldsymbol{x}}$ ± SEM		$\overline{\boldsymbol{x}}$ ± SEM		$\overline{\boldsymbol{x}}$ ± SEM	
	Poznań	Lviv	Poznań	Lviv	Poznań	Lviv
Number of subjects	50	58	25	33	25	25
Mean age in years	33.62 ± 1.90 (M = 28.5 ÷ U/L Q = 25/40) NS	39.26 ± 1.57* (M = 41 ÷ U/L Q = 30/46) *p* = 0.0242	35.92 ± 2.80 (M = 31 ÷ U/L Q = 25/46) NS	38.55 ± 2.52 (M = 42 ÷ U/L Q = 28/47) NS	31.32 ± 2.54 (M = 28 ÷ U/L Q = 26/29) NS	40.20 ± 2.55* (M = 40 ÷ U/L Q = 32/46) *p* = 0.0173
Average duration of hospitalization in days	54.64 ± 3.96 (M = 48 ÷ U/L Q = 30/75) NS	38.43 ± 2.18* (M = 37 ÷ U/L Q = 28/48) *p* = 0.0006	43.64 ± 4.44 (M = 37 ÷ U/L Q = 25/55) NS	41.18 ± 2.98 (M = 41 ÷ U/L Q = 34/48) NS	65.64 ± 5.85^+^ (M = 64 ÷ U/L Q = 45/87) *p* = 0.045	34.80 ± 3.08* (M = 35 ÷ U/L Q = 20/40) *p* < 0.0001
Person–days used	2,732	2,229	1,091	1,359	1,641	870

Source: based on results of our own studies

*M* median, *NS* non significant, *U*/*L Q* upper and lower quartile

*Statistically significant difference: Poznań vs Lviv for *p* < 0.05

^+^Statistically significant difference: women vs men for *p* < 0.05

The analyzed group of Polish patients hospitalized for a total number of 2,732 days generated costs of stay at the value of PLN 593.367 (EUR 145,790.42), corresponding to 90.79 % of the total calculated costs in Poznan (see Table 2). Women proportionally to hospital days used (1,091) generated a cost of PLN 245,475 (EUR 60,313.27), and men, of PLN 347,892 (EUR 85,477.15). The cost of hospitalization of patients from Lviv is UAH 301,583.5 (EUR 28,586.11), corresponding to 92.38 % of the total calculated costs in Lviv. Women’s hospitalization cost is UAH 183,872.5 (EUR 17,428.67), and men’s hospitalization cost is UAH 117,711 (EUR 11,157.44). These amounts point to the scale of financial resources allocated to provide effective schizophrenia treatment. Considering the fact that in Poland, approximately 30,000 people [15] are hospitalized annually in psychiatric hospitals and assuming the value of medical procedures and the values of hospitalization calculated in the study and then converting them to the number of people treated in psychiatric hospitals, the costs associated with the stay of patients in Polish hospitals are a burden on the state budget amounting to PLN 350 million.

**Table 2:** Treatment costs in Poznań and in Lviv (EUR)

	Total			Poznań			Lviv		
	Women	Men	Overall (% of total costs)	Women	Men	Overall (% of total costs)	Women	Men	Overall (% of total costs)
Hospitalization cost (% of total costs)	77,741.94 (40.59 %)	96,634.59 (50.46 %)	174,376.53 (91.05 %)	60,313.27 (37.55 %)	85,477.15 (53.24 %)	145,790.42 (90.79 %)	17,428.67 (56.32 %)	11,157.44 (36.06 %)	28,586.11 (92.38 %)
Pharmacotherapy cost (% of total costs)	5,325.21 (2.78 %)	7,256.23 (3.79 %)	12,581.44 (6.57 %)	3,949.32 (2.46 %)	6,643.88 (4.14 %)	10,593.20 (6.60 %)	1,375.89 (4.45 %)	612.35 (1.98 %)	1,988.24 (6.43 %)
Diagnostic tests cost (% of total costs)	1,701.83 (0.89 %)	2,855.65 (1.49 %)	4,557.48 (2.38 %)	1,511.06 (0.94 %)	2,677.40 (1.67 %)	4,188.46 (2.61 %)	190.77 (0.61 %)	178.25 (0.58 %)	369.02 (1.19 %)
Total cost	84,769.28 (44.26 %)	106,745.08 (55.74 %)	191,515.45 (100 %)	65,773.65 (40.95 %)	94,798.43 (59.05 %)	160,572.08 (100 %)	18,995.33 (61.38 %)	11,948.04 (38.62 %)	30,943.37 (100 %)

Source: based on results of our own studies

The pharmacotherapy cost of patients treated in Poznan was PLN 43,114.33 (EUR 10,593.20), corresponding to 6.60 % of the total calculated costs, and in Lviv, it was UAH 20,975.93 (EUR 1,988.24), corresponding to 6.3 % of the total calculated costs. In Poznan, women’s pharmacotherapy cost was PLN 16,073.72 (EUR 3,949.32), and men’s pharmacotherapy cost was PLN 27,040.61 (EUR 6,643.88). In Lviv, these costs were women UAH 14,515.68 (EUR 1,375.89) and men UAH 6,460.25 (EUR 612.35).

**Fig. 1:**
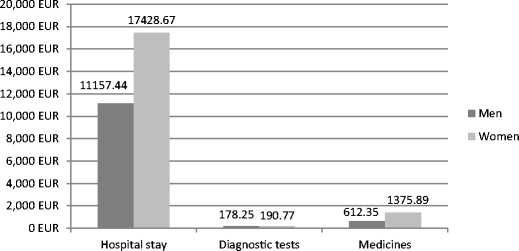
Cost of the inpatient care of schizophrenia in Lviv (Euro). Source: based on results of our own studies

**Fig. 2:**
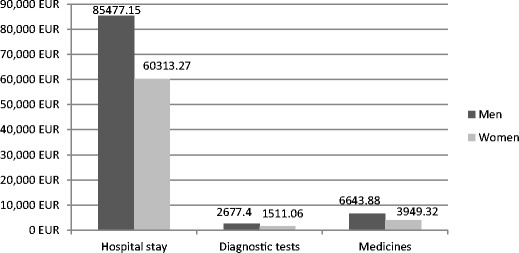
Cost of the inpatient care of schizophrenia in Poznan (Euro). Source: based on results of our own studies

Diagnostic tests in Poznan accounted for 2.61 % and in Lviv for 1.19 % of the total costs (Figs. 1 and 2). In Poznan, the cost of diagnostic tests for women was PLN 6,150 (EUR 1,511.06) and for men, PLN 10,897 (EUR 2,677.40). In Lviv, it was UAH 2,012.71 (EUR 190.77) for women and UAH 1,880.53 (EUR 178.25) for men.

The total cost of treatment of 50 patients with schizophrenia (hospitalization + pharmacotherapy + diagnostics tests) in Poznan was PLN 653,528.33 (EUR 160,572.08), and per patient it was PLN 13,070.57 (EUR 3,211.44). In Lviv, the total cost of treatment of 58 patients was UAH 326,452.55 (EUR 30,943.37), and per patient, it was UAH $\overline{x}$ = 5,628.5 (EUR 533.5).

Interesting data come also from the analysis of the prescribed pharmacotherapy. Patients in Poznan were treated with all generations of antipsychotics. Of other medicines, olanzapine was administered to 64 % of patients, long-acting risperidone to 12 %, and aripiprazole to 32 % of patients. In Lviv, patients received mainly first-generation antipsychotics or older atypical neuroleptics, whereas olanzapine, long-acting risperidone, and aripiprazole were administered to 1.72, 0, and 0 % of patients, respectively. Haloperidol was the most popular medicine in Lviv, and 36.21 % of patients obtained this neuroleptic. In Poland, patients were likewise treated with haloperidol, but each patient obtained atypical neuroleptics concurrently. In Lviv, some patients were treated only by haloperidol or in combination with different typical neuroleptics like chlorpromazine, which was not used in Poznan. Among newer neuroleptics, the most frequently used medicines in Lviv were clozapine (36.21 % of patients) and risperidone (17.20 % of patients).

Apart from the analysis of pharmacotherapy used, the appraisal of the frequency of schizophrenia relapses within the last 10 years provides interesting data. In the range between 0 and 3 hospitalizations, there were 23 patients in Poznan and 12 in Lviv, whereas in the range from 4 to 7 inpatient stays, there were 11 patients in the Polish center, comparing to 19 patients from Lviv. In a category above seven hospitalizations within the last 10 years, a significant difference was observed in both analyzed centers. In Poznan, there were 16 patients, but in Lviv there were 27 patients.

## Discussion

The calculation of costs associated with schizophrenia may significantly differ among countries. There are still a few, especially concerning low-income countries, pharmacoeconomic analyses presenting a common trend of results obtained by different research groups. The main reason of discrepancies in the analogous analyses can be economic availability of modern pharmacotherapy to patients, the lack of support in the form of non-pharmacological therapy for patients and their families, and, above all, different health care systems and different state budget expenditures for treatment of mental disorders. In Ukraine, patients in many cases have to buy modern medicines, also during hospitalization, on their own. The original brand of olanzapine (5 mg × 28 tablets) costs approximately EUR 100 in the Ukraine and, like long-acting risperidone, which in Ukraine costs between EUR 125 and 180, is not reimbursed there. In Poland, patients receive these medicines for free during hospitalization. In outpatient care and in accordance to the notice of the Polish Minister of Health of December 21, 2012, these medicines cost, after reimbursement, EUR 0.79 (long-acting risperidone 37.5 mg × 1 syringe) and EUR 10.74 (original olanzapine 5 mg × 28 tablets). Moreover, in addition to the differences in price and in the way of health care funding, Ukrainian citizens have significantly limited economic access to pharmacotherapy compared to Polish patients. It is related to the average earnings of residents of the analyzed countries. In Poland, in January 2012, the average salary was approximately net to EUR 660, and in Ukraine, it was about EUR 225. Because of the system of funding and the organization of psychiatric care, patients in Poland are treated with a wider range of atypical neuroleptics. However, due to economic reasons, Ukrainian patients are obligated to use older antipsychotic medicines. Some studies have indicated that older neuroleptics may be as effective as atypical antipsychotics. The CATIE study conducted by Lieberman et al. [22] showed that there were no relevant discrepancies in efficiency between the first-generation drug perphenazine and newer medicines. However, it seems that there are also some limitations of the CATIE study, including the limited amount of conventional medicines taken into consideration and relatively low dosing of medicines used [23]. Moreover, Jarema et al. found better efficacy and safeness of atypical olanzapine in relation to the perphenazine [24]. The CATIE study has not included medicines like haloperidol, which in the opinion of the authors of CATIE, is rarely used. However, presented results and the analysis conducted by Daltio et al. [12] show that haloperidol is still a frequently used medicine. In spite of the fact that new antipsychotics are comparable in efficacy to older brands of neuroleptics, their better tolerability and less extrapyramidal side effects [5, 24] can contribute to the better compliance, which is an important factor of rehospitalization rates and costs of inpatient care concurrently [25]. Although the use of atypical antipsychotics could result in the current increase of the total cost of schizophrenia treatment, their effectiveness may pay off in fewer side effects of pharmacotherapy, better compliance, and reduced hospitalizations. Therefore, more expensive drugs could be cost-effective for society in the long term [5, 7]. Such results could also contribute to the more effective teaching of residents, providing them food for thought about the importance of economic considerations and the cost of inpatient care and proper funding of many dimensions of the treatment of schizophrenia.

The lack of reimbursement system, limited funds of Ukrainian patients and their families, and significantly higher prices of atypical neuroleptics in comparison to the Polish pharmaceutical market are probably the main reasons of the observed discrepancies in the number of patients treated with olanzapine, long-acting risperidone, and aripiprazole. Despite this, the observed differences in relation to costs of schizophrenia treatment in Poznan as compared to Lviv are not a surprising exception. In the analysis conducted in six European countries, discrepancies in the cost of treating patients suffering from schizophrenia were also presented [26], for example, with a 12-fold discrepancy between centers in Spain and in Switzerland. Therefore the sixfold difference between Poznan and Lviv is not alarming, because the percentage of each component of the total cost was similar in the analyzed centers, and the discrepancy is quite proportional to the dissimilarity in the cost per day in both cities. As in other studies [27], in our study the difference was mainly related to the different organization and funding of medical care, and therefore results of some research could differ significantly.

In France, Rouillon et al. assessed that the total direct costs of schizophrenia treatment were FRF 12 billion [7, 28]. In the opinion of Meerding et al., costs of schizophrenia treatment in the Netherlands amounted to NLG 800 million [7, 29]. The study of Goeree et al. demonstrated that direct costs of schizophrenia amounted to 1.12 billion Canadian dollars versus 2.4 billion of total costs spent on the treatment of schizophrenia [7, 30]. It is estimated that the costs of medicines used in patients with schizophrenia in developed countries (Germany, Italy, Canada) amount from 1 to 9 % of total direct costs [1, 7]. Results obtained in our own study confirm this trend. In Poznan, pharmacotherapy accounted for 6.60 %, and in Lviv, 6.43 % of total costs. However, the study of Suleiman et al. conducted in Nigeria showed that costs of antipsychotic medicines accounted for 53 % of total direct costs [7, 31].

Our study confirms the importance and profitability of investment in innovative and comprehensive treatment. Patients treated in Poznan with the use of a wider range of innovative neuroleptics and covered by more widespread treatment in comparison to patients from Lviv were characterized by fewer relapses of schizophrenia within 10 years since the beginning of the analysis. Therefore, in spite of the single amelioration in expenditures related to the embracement of the broadest amount of patients with modern and complex therapy, it will contribute to the reduction of hospitalizations and simultaneously costs of schizophrenia in the long run.

Such analyses and obtained results confirm the need of education of residents, who will be aware of clinical and economic facets, during times of limited resources that determine an expected improvement and development of the treatment of mental disorders. Thus, residents ought to take part in additional courses concerning costs in mental health, and results of economic analyses should encourage authorities responsible for developing curricula and syllabuses to the implementation of updated educational programs that are aimed to provide useful and innovative knowledge. During lectures or clinical courses, residents should be taught about the economic differences between neuroleptics also in relation to the cost-effectiveness of used medicines and non-pharmacological treatment. It will contribute to the awareness of future clinicians or health care decision makers about the importance of comprehensive treatment, which in spite of the inevitable investment, will lead to significant savings in the long run. Besides, residents should be presented with the knowledge about the cost of inpatient care from hospital records. Therefore, it seems interesting to introduce in hospital records obligatory information about general costs of inpatient care and about individual components, like cost of pharmacotherapy. It will allow residents to compare both clinical and economic effectiveness of applied therapy.

The increase of expenditures on modern pharmacotherapy in Ukraine, for instance, by a wider availability of innovative medicines and by reimbursement of atypical neuroleptics, could contribute to the reduction of total costs of schizophrenia treatment in the long term. Experts believe that access to innovative neuroleptics must not be a privilege—it should be a right for every single patient [1]. The cost-effectiveness of using atypical neuroleptics is confirmed by results of pharmacoeconomic analyses, which indicate that therapy with innovative medicines is generally cheaper than therapy with older generations of antipsychotics [32, 33]. It guarantees fewer hospitalizations and the cost reduction of side effects related to treatment with old neuroleptics [1]. In spite of the huge economic burden associated with schizophrenia treatment, costs can be reduced by implementing solutions like psychoeducation, which contributes to the improvement of treatment efficacy. Psychoeducation can also lead to reduction of rehospitalization rates and can help both patients and their families to cope with the mental illness in a more effective way [25]. Apart from that, non-pharmacological interventions lead to the improvement and support the concept of comprehensive treatment also related to early intervention in psychotic disorders [34]. Such trainings in community care are also a great chance for a better social functioning of people with mental disorders and for their successful comeback to the labor market. Ergotherapy can lead to cost-effective treatment results in the long run [1]. Therefore creation of sheltered job centers should be a kind of a priority in a mental health policy in Poland and Ukraine. Despite many advantages, there are still few job centers in Poland. However, in Ukraine, such community psychiatry almost does not exist, mainly due to the lack of governmental financial support.

In the study conducted by Rummel-Kluge et al. [25], the authors found that an increase in the number of patients included in such non-pharmacological therapy can contribute to the reduction in the duration of hospitalization, and as a result of saved hospital days, it can contribute to the reduction of direct costs by EUR 150 million [25]. Significant savings can be expected also in Poland and in Ukraine. As it is presented in Figs. 1 and 2, the cost of the patient’s hospital stay is the most important burden in the group of direct costs. Therefore, every effort leading to a reduction in the duration of hospitalization should be treated as a priority. Psychoeducation, as a cost-effective part of a comprehensively understood treatment of mental disorders, is presented also by Shimodera et al. [35]. Fewer hospitalizations also create a chance for even partial recapture of productivity among patients and their health caregivers who, according to an analysis by Chang et al. [11], were responsible in South Korea for indirect costs amounting to US$ 132 million and related to the productivity loss [11]. It should be pointed out that apart from the source of income, work for patients during remission has an important psychological function and affects the course of their disorder. Studies confirm the correlation of patients’ social condition deterioration with a more severe course of schizophrenia [1, 36]; therefore, every form of protected employment should contribute to a reduction of schizophrenia costs and to a better functioning of patients suffering from schizophrenia in the psychosocial dimension.

Governments of every country and people responsible for the budgets allocated for mental health should strive to increase financial outlays for modern and comprehensive treatment of mental disorders. In a long-term perspective, the invested money will benefit in significant savings. Besides, patients who require rarer and shorter hospitalizations enjoy a better quality of life, they are characterized by an easier return to social functions performed before the illness, and they are more successful in seeking solutions that allow them to regain their productivity lost due to the illness. Such an image is consistent with the strategy developed by WHO experts [2], who emphasized the need for a holistic approach promoting mental health, preventing mental disorders, and limiting the social stigma of a person with psychiatric disorder.

The present study has a few limitations: the time horizon of the study could be extended, and the sample size could be larger. Inclusion of younger patients as well could lead to an interesting comparison of pharmacotherapy schedules and their efficacy in teenaged and adult groups of patients. The analysis of indirect costs could be an interesting development of such research. Although Poland and Ukraine have different health care systems and there are economic discrepancies between these countries, this study is an interesting source of data concerning treatment schedules and costs of inpatient care of schizophrenia. The study concerns also drawbacks of the health care system in both academic centers; thus, residents from these centers will consider future pharmacoeconomic facets of therapy both from academic and clinical perspectives and in potential perspective of people responsible for effective mental health care. However, further studies are still necessary for a better estimation of schizophrenia. Conducting such analyses in academic hospitals will allow academic leadership, and these hospitals could be a source of governmental support, due to reliable results of studies conducted in academic centers. On that basis, graduated residents as future health care decision makers, clinicians in smaller hospitals, or lecturers of subsequent generations of pupils could apply this unique knowledge.

Schizophrenia generates huge direct costs, which differ between study centers in particular because of different systems of mental health care funding. In Poznan, cost of inpatient care turned out to be six times higher than in Lviv. Despite this fact, the percentage of each component of the total cost was similar in the analyzed centers. Medicine prices significantly differ between the analyzed countries, and thereby, economic availability of a specific pharmacotherapy can be limited and can affect direct and indirect costs and patients’ quality of life. Comprehensive treatment is cost effective, and patients treated with a wider range of atypical neuroleptics and with more severe non-pharmacological treatment were less hospitalized. Economic availability of innovative and effective treatments should be equalized by actions aimed at lowering drug prices or their adjustment to the patients’ affluence. Moreover, innovative and cost-effective pharmacotherapy should be reimbursed because it should provide a more effective health care also in economic terms. Although investment in the innovative and comprehensive treatment could ameliorate a critical part of the costs of schizophrenia, it would be cost-effective in the long run nonetheless.

**Table:** 

Implications for Educators
• Academic educators are responsible for interdisciplinary teaching of residents to be thoughtful also about the economics and cost of inpatient care.
• The introduction of mental health economy to educational programs and curricula will allow residents to acquire suitable experience at the academic level and contribute to awareness about the importance of analyses of cost of inpatient care and about the ways of cost-effective treatment of schizophrenia in the long run.
• Academic hospitals could be a leader of the development and spreading of cost-effective therapeutic solutions that could have important implications for funding of psychiatric care and for residents who will be responsible for a profitable treatment.
